# Reactions of roe deer to human disturbances and wolf predation in a Polish forest

**DOI:** 10.1093/cz/zoaf059

**Published:** 2025-08-28

**Authors:** Haidt Andżelika, Gawryś Radosław, Szewczyk Maciej, Kateryna Lipińska, Karolina Lubińska

**Affiliations:** Department of Forest Ecology, Forest Research Institute, Braci Leśnej 3, Sękocin Stary 05-090, Poland; Department of Forest Ecology, Forest Research Institute, Braci Leśnej 3, Sękocin Stary 05-090, Poland; Department of Vertebrate Ecology and Zoology, Faculty of Biology, University of Gdańsk, Wita Stwosza 59, Gdańsk 80-308, Poland; Department of Forest Ecology, Forest Research Institute, Braci Leśnej 3, Sękocin Stary 05-090, Poland; Bory Tucholskie National Park, Długa 33, Charzykowy 89-606, Poland

**Keywords:** fear ecology, gray wolf, hunting, recreation, roe deer

## Abstract

Antipredator responses of prey are important because of their potential trophic cascade effects. Predator responses are especially important for species that impact the economy, such as ungulates. Past research has focused on the behavioral adaptation of roe deer, the most common ungulate in Europe, to human and lynx pressure. However, the European wolf population has increased in the last decade and in some recently recolonized parts of Poland roe deer constitute the largest food source for wolves. Unlike in moose and red deer, no evidence has yet been found for changes in the vigilance-foraging time ratio in roe deer in response to wolves. We took the unique opportunity to study the behavior of roe deer during the partial recolonization by wolves of a large forest complex (Tuchola Forest) in Poland. Using video cameras, we found that roe deer react differently to the presence of wolves compared with humans. Wolf predation increases roe deer movement. In comparison, anthropopressure causes roe deer to mainly use an avoidance strategy, limiting their activity to times of day when people are not present. This temporal avoidance strategy was similar whether human pressure resulted from recreation or hunting.

## Graphical Abstract

**Graphical Abstract:**
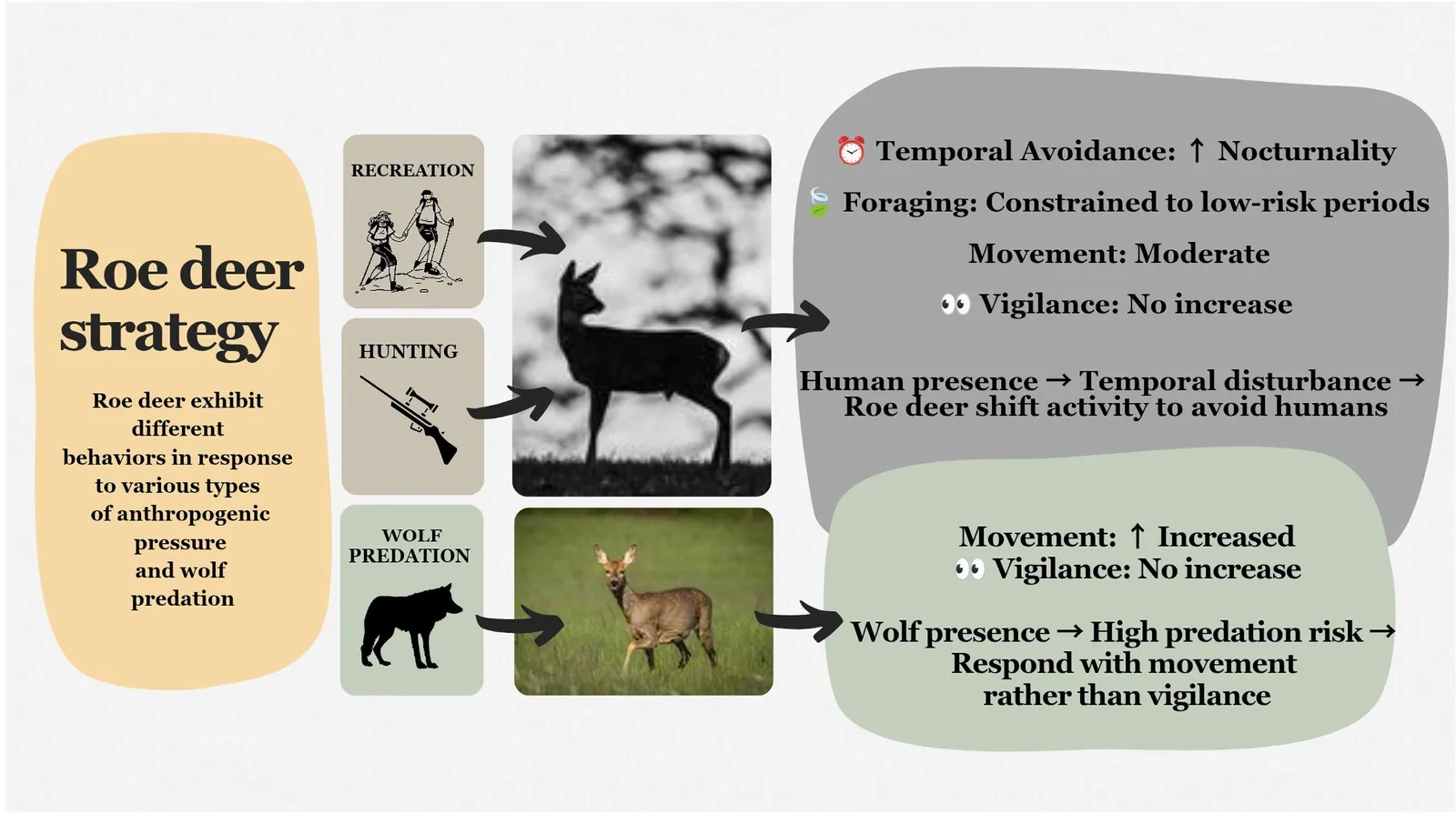

The central European roe deer (*Capreolus capreolus*) population is estimated to be 15 million and increasing (Lovari et al. 2016; Linnel et al. 2020). In Poland its population also has been increasing steadily in recent years. This growth is attributed to several factors, including, for example, effective wildlife management, favorable habitat conditions (Hušek et al. 2021). It occupies a wide variety of habitats, from forests to urban areas (Jasińska et al. 2021). Roe deer are managed primarily by hunting to reduce population densities (Burbaite and Csányi 2009), but nonlethal effects of hunting are also known to change the behavior of roe deer (Benhaiem et al. 2008; Bonnot et al. 2013; Picardi et al. 2019). Human presence during hunting is one of the most important factors affecting deer behavior, along with the effects of recreation and of predation by lynx (*Lynx lynx*) (Bonnot et al. 2013, 2020; Eccard et al. 2017; Carbillet et al. 2020). Along with lynx, predation by wolves influences ungulate populations and behavior (Creel and Christianson 2009; van Beeck Calkoen et al. 2018; Mpemba et al. 2019; Ausilio et al. 2021; Raynor et al. 2021; Loosen et al. 2021).

The gray wolf (*Canis lupus*) was at one time extirpated from most of Central Europe. As a result of conservation measures, wolves have recolonized much of their historical range (Chapron et al. 2014). The reestablished Central European wolf population is now rapidly spreading further west in Europe from western Poland (Szewczyk et al. 2021). The increase in the wolf population in Poland has been closely linked to the growing populations of ungulates such as roe deer, which are a primary part of the wolf's diet (Nowak and Mysłajek 2016; Rodríguez-Recio et al. 2022). Most wolf-ungulate studies have been conducted in North America and focus on wolf impacts on elk (*Cervus elaphus canadensis*). The presence of wolves leads to significant behavioral changes in elk, including increased vigilance and reduced foraging activity, which negatively impacts their physical condition (Creel and Winnie 2005). Hebblewhite and Merrill (2007) found that wolves alter elk migration patterns, with the magnitude of this effect depending on habitat availability and the seasonal dynamics of predator populations. In the long term, wolves also cause a decrease in elk numbers and changes in vegetation structure due to reduced grazing pressure (Halofsky and Ripple 2008; Laundre et al. 2010). Return of predators can lead to significant changes in prey populations and ecosystem dynamics (Loosen et al. 2021). Wolf predation has an impact on ungulate behavior and vegetation in European forests, finding that wolves induce changes in prey movement and foraging patterns (Kjupier et al. 2014). Proudman et al. (2021) analyzed the cascading effects of predators on biodiversity, highlighting the role of apex predators in shaping ecosystem structure. Predator presence has an impact on prey stress levels, showing that predation risk alters prey physiology (Van Beeck Calkoen et al. 2018) The best-studied effect of the wolf on the behavior of roe deer was described in studies from southern Europe (Bongi et al. 2008; Rossa et al. 2021). Changes described in the literature in roe deer behavior were temporal, spatial and physiological (Gerber et al. 2024). Roe deer exhibited minimal temporal avoidance of wolves compared with other ungulate species (Rossa et al. 2021). Instead, wolves adjusted their activity patterns to align with those of roe deer, rather than roe deer altering their activity to avoid wolves. We believe that the absence of reports of wolf impacts on proportion of vigilance to foraging is due to lynx (the main predator of roe deer) also being present in the area where those studies were conducted and that the population density of roe deer was low (i.e., sample sizes were too small to discern significant results) (Kuijper et al. 2013). However, in some parts of Central Europe, roe deer constitute a major component of wolf diets. For example, in Poland, they were the main prey of wolves in the western part of the country immediately after wolves recolonized this region, where it comprised 42% of the wolf diet (Nowak et al. 2011). The intense predation pressure from wolves on the roe deer population may have triggered strong behavioral responses in this area—such as increased vigilance or reduced foraging—that have not previously been documented in this species as part of its antipredator strategies toward wolves (Gerber et al. 2024).

Humans as super predators can also profoundly change deer activity patterns throughout the day (Bonnot et al. 2020). Recreation is one form of anthropogenic disturbance affecting ungulate behavior. A study in the Netherlands showed that roe deer avoided areas with high recreational activity, leading to reduced sapling browsing on open heathland, while hunting had no additional effect on their behavior (Mols et al. 2022). Red deer in the Białowieża Forest respond differently to the presence of humans and wolves; they show increased vigilance in protected areas, likely due to higher wolf activity in those parts of the forest. Moreover, during the hunting season, deer are more vigilant during daytime, outside protected areas, that is, at the time and place where hunters are active (Proudman et al. 2021). In contrast, a study conducted in the Bavarian forest (Germany) suggests that the effects of human activity on deer outweigh those of a large predator, the lynx (van Beeck Calkoen et al. 2022).

Areas partially occupied by predators (e.g., during recolonization of an area by a predator) can be used to analyze prey behavior over time in similar environmental conditions. Such a unique situation occurred in Poland while conducting research for the current investigation. Tuchola Forest has distinct feeding and shelter areas: meadows, where foraging takes place, and young pine forests, where cover is available. High visibility in meadows probably increases deer avoidance behavior because it increases detectability by predators and decreases escape opportunities. Cover, on the other hand, is crucial for protection from predation by humans and wolves. However, wolves also choose places with good cover for their bed sites (Bojarska et al. 2021).

Visibility in context of roe deer vigilance, foraging and time spends on movement has never, to our knowledge, been the focus of research as a factor affecting roe deer behavior. The aim of this study was to determine how roe deer behavior changes during partial recolonization of a large forest complex (Tuchola Forest) by wolves. We hypothesized that roe deer react differently to human pressure and wolf predation. The first hypothesis assumes that roe deer, like most ungulates, temporarily avoid areas of greatest human activity. Roe deer may shift their activity patterns becoming more nocturnal or crepuscular to reduce encounters with humans and wolves. The second hypothesis assumes that roe deer respond to wolf pressure with increased vigilance. We believe our work is an important starting point for other studies of roe deer–wolf interactions, which is important because the wolf is the most numerous large carnivore in Europe, and the roe deer is the most numerous ungulate, and both species have major economic impacts.

## Materials and methods

### Study area

The Tuchola Forest (Bory Tucholskie) is located in northern Poland in Central Europe, primarily in the Pomeranian Voivodeship. It lies south of Gdańsk and north of Bydgoszcz, between the Brda and Wda rivers. The region is characterized by vast pine forests, lakes, and sandy soils. Tuchola Forest is one of the largest forest complexes in Poland. It occupies about 3,000 km² (Kondracki 2002). Over 90% of the area is covered by forests, with the remaining 10% consisting of meadows and pastures, arable fields and buildings. Scots pine (*Pinus sylvestris*) is the dominant tree species in forest stands.

**Figure 1:**
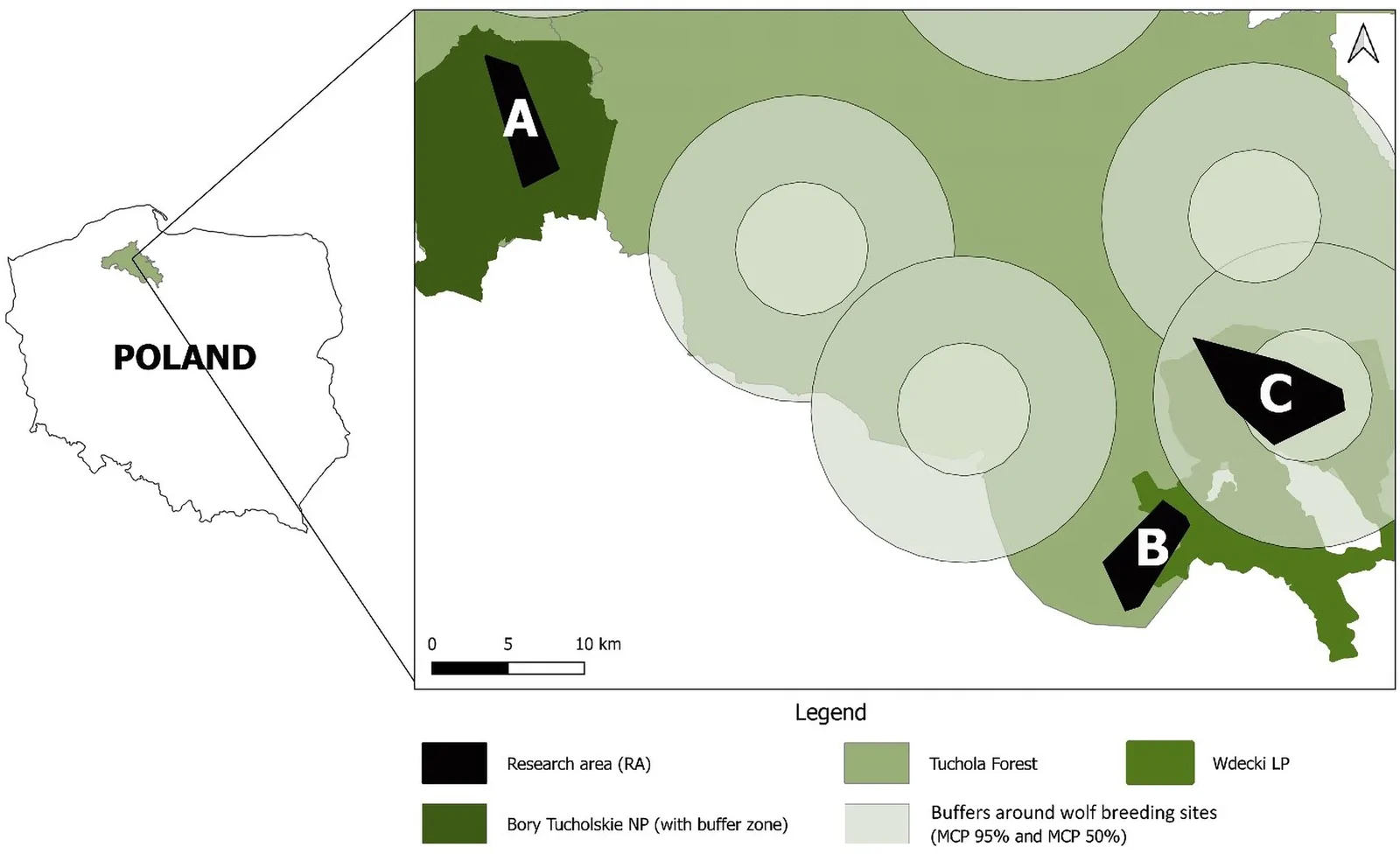
The location of the Tuchola forest in Poland and the locations of research areas (A, B, and C). Wolf territories are presented schematically as circle buffers around reproduction sites (dens/rendezvous sites with pups). Buffer sizes are based on telemetric data from a similar study area in Western Poland (Drawa Forest, Mysłajek et al. 2018). The 60 km^2^ buffers represent packs’ central territories and correspond to minimum convex polygon (MCP) 50% from the aforementioned paper, while 310 km^2^ corresponds to MCP 95%.

Three research areas were established in Tuchola Forest. Each area had similar habitat conditions and various levels of hunting pressure, recreational use and wolf predation risk (Fig. 1).

**Figure 2:**
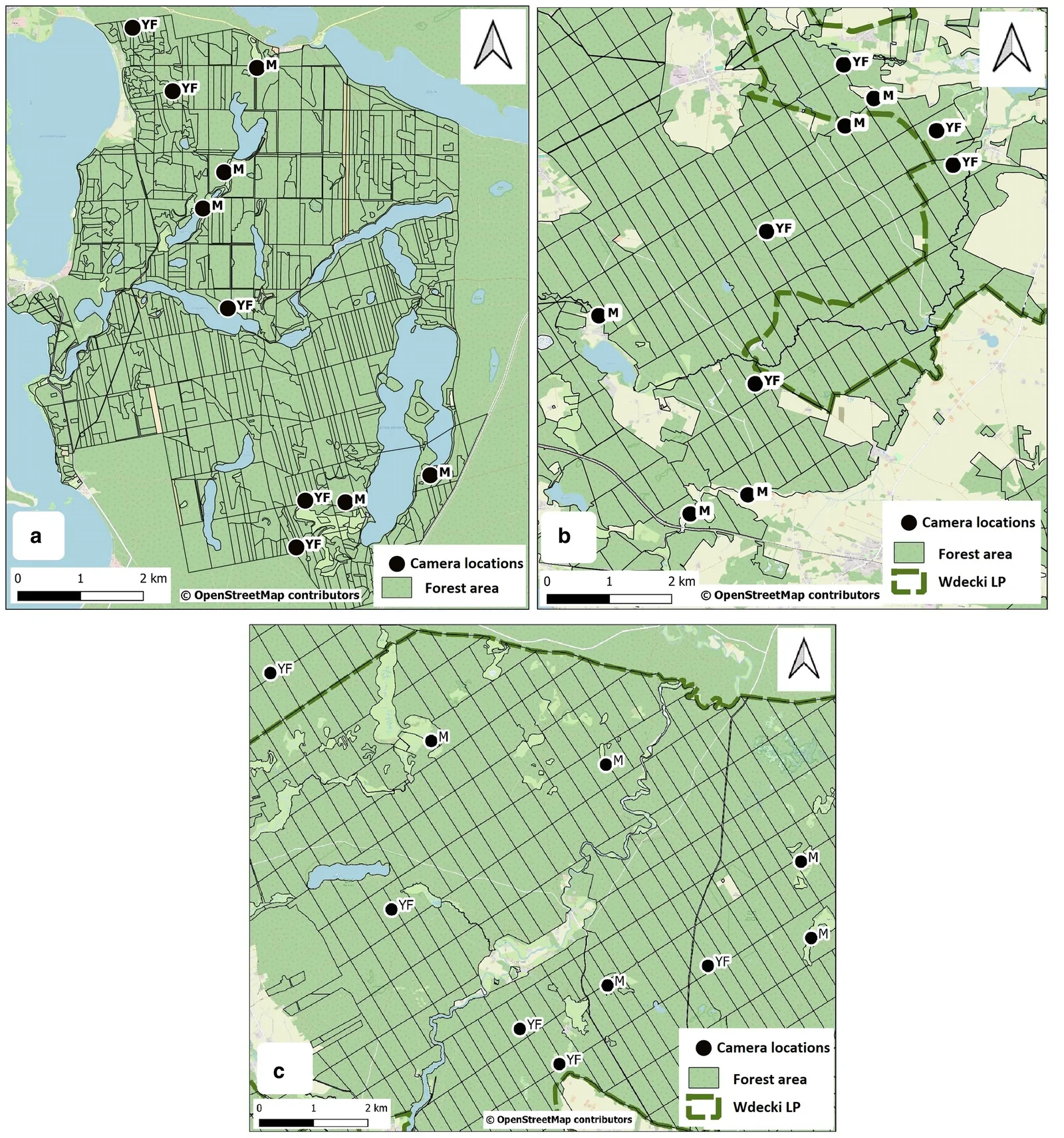
Camera locations (M—meadow *n* = 5; YF—young forest *n* = 5) in research area (a): BTNP, (b): Zamrzenica and Trzebciny forest districts, and (c): Trzebciny and Osie forest districts.

- Area A was established in the Bory Tucholskie National Park (BTNP), the area furthest west covering 46 km^2^ (Fig. 2a). Coniferous forests dominate, occupying almost 98% of the park's forest area. The BTNP is the only national park in Poland where it is possible to pick mushrooms and berries (i.e., it has off road use and contains hiking trails). In this area, there is no hunting season for roe deer since 2005, so the area is completely free of hunting pressure for this species (data from the National Park). Population of roe deer was estimated on 17.6 individuals per 10 km^2^ and no hunting was carried out on this species during the research. At the beginning of the study, there was no wolf family group in this part of the park and no confirmed reproduction according to published data (Nowak et al. 2017). Wolf monitoring results confirm that until winter 2016/17, only sporadic occurrence of dispersing wolves was detected. Estimated data on the number of tourists visiting the Park are 35,200 in 2016 and 31,700 in 2017 (data from Bory Tucholskie National Park).
- Area B, with a size of 37 km^2^, is located on the border between Zamrzenica and Trzebciny forest districts (Fig. 2b). Game management in this area is through the state forests, and hunting is intense. At the beginning of the survey, there was neither permanent wolf presence nor confirmed reproduction in the area (Nowak et al. 2017). However, we detected sporadic presence of wolves from a pack which had its central territory about 20 km north-west of the study area (Fig. 1), likely representing extraterritorial movements associated with hunting and/or natal dispersal. The roe deer population, according to Forest Database (https://www.bdl.lasy.gov.pl/portal/tworzenie-zestawienia-rlo), was 70 individuals per 10 km², while the number of roe deer killed by hunters in 2017 (a measure of human hunting pressure) was 12.4 per 10 km^2^. Area B is located on the outskirts of the BT forest complex, closer to human settlements compared with Area C, which lies deeper within the forest. Several villages, such as Wierzchucin, Zdroje, and Jelenia Góra, are situated within Area B. Unlike the tourist-attractive national park, Area B is neither protected nor promoted, making it less intensively used for recreation. As a result, the level of recreational activity in Area B falls between that of Areas A and C.
- Area C has an area of 50 km^2^ and is on the border of the Trzebciny and Osie forest districts and includes part of the Wdecki Landscape Park (Fig. 2c). In this area, only individual hunts are conducted by the Polish Hunting Association. There is a wolf family group with confirmed reproduction in this area (Nowak et al. 2017), present there at least since 2012. Wolf pups were detected within the study area during the study, as well as in the preceding and following years, indicating that the area constitutes core part of the local pack's home range (Fig. 1). The roe deer population was 34.8 individuals per 10 km^2^, while the number of roe deer killed by hunters in 2017 was 0.8 per 10 km^2^. Area C is located far from villages, deep within the BT forest complex.

We assume randomness of recreational activity and hunting in both types of habitats: meadows and young forest.

According to the literature, the European lynx does not occur in the research areas (Mysłajek et al. 2019).

The maps of the distribution of the research areas and the camera traps were created in QGIS 2.18.2 Las Palmas using an OpenStreetMap background (© OpenStreetMap contributors, available under the Open Database License, ODbL 1.0). Shapefile layers provided by the Tuchola Forest National Park and the forest districts of Osie, Trzebciny, and Zamrzenica were used to visualize the forest areas, with permission from the respective authorities.

### Wolf monitoring

Wolf packs inhabiting Tuchola Forest have been monitored since 2012 in a long-term monitoring effort conducted by Association for Nature “Wolf,” University of Warsaw and University of Gdańsk and coordinated by one of coauthors of this study (S.M.). Core territories of wolf family groups were identified using a combination of methods, including winter snow tracking surveys (performed each year mostly in January and February), searches of pup presence signs in summer (performed each year from ca. 15 July to 15 September), year-round camera trapping and DNA analyses of samples collected mostly during winter monitoring.

Wolves were tracked by foot, by bike or by car, using the dense and regular network of forest roads in the study area. Searches for the presence of tracks, scats or ground scratches were focused mostly on road junctions, which are used by wolves as scent-marking hotspots (Stępniak et al. 2020). Additionally, signs of pup presence such as tracks and various objects used as toys by juvenile wolves (for details, see Ausband 2021) were looked for in late summer. In identified rendezvous sites and scent-marking hotspots camera traps were installed. During each inspection, attention was also given to the potential signs of lynx presence.

Noninvasive DNA samples (scats, hair, urine, and estrus blood from snow) were collected during fieldwork and processed in a dedicated genetic laboratory as described before (Szewczyk et al. 2019). Samples were genotyped in 13 unlinked autosomal microsatellite markers used previously to study wolf population genetics (Szewczyk et al. 2019; Stępniak et al. 2020). To distinguish between family groups and assess relatedness of individuals, individual microsatellite genotypes were analyzed using combination of methods: 1) calculation the pairwise Lynch and Ritland (1999) relatedness estimator in GenAlex (Peakall and Smouse 2012), 2) manual comparison of related genotypes in Microsoft Excel, and 3) identification of kin groups with software dedicated for parentage analysis based on codominant genetic markers: Colony (Jones and Wang 2010) and Cervus (Kalinowski et al. 2007). Estimation of the number of wolf family groups and of their approximate home ranges was based on the total monitoring effort of 60 tracking days during 2 monitoring seasons, resulting in collection of 134 DNA samples and finding of 249 other wolf presence signs (mostly old scats and tracks). Specifically, in the wolf monitoring season preceding our study (2015/16), monitoring effort included 27 tracking days, analysis of 56 genetic samples and 89 other wolf presence signs, while during the study period (season 2016/17) we tracked wolves for 33 days, resulting in finding of 78 DNA samples and 160 other signs. DNA analyses were performed to confirm or exclude the presence of wolf family groups in the study areas. This was necessary to classify the risk of wolf predation. Genetic analyses served as a complement to the camera trap method.

### Camera traps

Camera traps have previously been used to conduct behavioral analyses and to determine the activity of roe deer throughout the day. Camera traps are a popular tool for studying wildlife ecology (Thomas 2011). They are noninvasive and do not require large financial resources (Tobler et al. 2008). Using the QGIS program (QGIS Las Palmas 2.18), ecosystems in the study areas were categorized as: young forest or meadow. Cameras were equally distributed between both habitat types in each area. Ten camera traps were placed in each study area (Fig. 2a–c). Camera traps were placed near ungulate tracks, droppings and feeding marks. Acorn KAM3 cameras with built-in infrared sensors were used. Camera traps were installed on tree trunks at the height of ∼0.5 m aboveground. The camera traps were oriented as far north as possible so that there were no heat sources (e.g., rocks that could be heated by the sun and activate the infrared sensor) and no sources of movement from, for example, protruding branches or tall grass, within camera range. Camera traps settings for the above recordings included the following parameters: lens viewing angle: 52°, resolution: 1440 × 1080 720p, video length of 20 s, and pause time between shots of 2 min.

Behavioral analysis of roe deer was performed based on recordings from 2 types of environments, distinguished by their level of cover: mid-forest meadows (a feeding site with high-predation risk [Torretta et al. 2018]) and young forest areas (refuge site).

Video recordings from camera traps were systematically controlled at 2–3-month intervals. During the growing season, when vegetation grows rapidly, camera traps in meadows were checked monthly to avoid being obscured by growing grasses or other vegetation. Videos recorded on memory cards were sorted into appropriate folders (i.e., wolf, human, roe deer) with information noted on date, season, time of day, environment (young forest or meadow), sex, and number of individuals.

Video capture rates for wolves, humans and roe deer were estimated for each study area based on phototrap recordings. For the research, we selected the time period during which all of them were operating simultaneously. The analysis was conducted for videos recorded from 11 August 2016, to 11 August 2017, during which all 10 camera traps in each area operated continuously (over a total of 365 video days). Subsequently, videos of wolves, humans, and roe deer were counted. This provided reliable data on the presence of wolves in study areas. Roe deer behavior was classified in each recording as vigilance, foraging, movement and other (Table 1), based on Winnie and Creel (2007) . To eliminate the pseudo replications, when the same camera trap recorded more than one video of the same individual within a 30-minute interval (Torretta et al. 2017), the sequence of recordings was considered as a single event. For further analysis, the time and date of the first recording in the series were used. Detections occurring within 1 h before sunset were categorized as dusk, and detections within 1 h after sunrise were categorized as dawn. The time of day was precisely and individually determined for each video based on data from the Institute of Meteorology and Water Management. At each site, the proportion of events was calculated for each of the 4 daily activity periods: dawn, dusk, day, and night, providing a comprehensive view of the animals' temporal activity patterns. This classification can be based on specific sun angles relative to the horizon, which helps in objectively distinguishing these periods in video analysis. The seasons are classified according to the calendar.

**Table 1.** Classification of roe deer behavior.

Behavior	Description
Vigilance	The animal stood still with its head held parallel to the body or higher, looking around and/or pricking its ears. Sniffing without chewing was also classified as vigilance
Foraging	Includes grazing (consuming grass or herbaceous vegetation) and browsing (consuming woody species)
Movement	Running and walking
Other	This included all other types of behavior, such as scratching or rubbing against trees, checking the phototrap (walking toward the camera, smelling it)

The classification includes the following types of behavior and their description: vigilance, feeding, movement, and other based on Winnie and Creel (2007).

### Statistical methods

The coefficient of overlap (Δ) was used to measure the degree of similarity between the activity curves of the species under study, ranging from 0 (no overlap) to 1 (identical curves). This is a crucial measure for comparing temporal activity patterns across species (for roe deer, wolves, and humans). Activity curves were determined for each 24-h daily period, and the time of each recording was converted to radians. Odds ratios (ORs) were reported to quantify the strength of association between variables, along with confidence intervals (CIs) to indicate the precision of the estimates. Overlap was calculated using the “overlapEst” function from the “overlap” package (Ridout and Linkie 2009). The 95% CIs were estimated using the “bootstrap” function (“overlap” package) with 1000 bootstraps and the “BootCI” function—value “basic0” (“overlap” package). We used the Δ1 overlap coefficient when sample sizes <50 and Δ4 for samples >50.

Watson–Wheeler test was used to compare the homogeneity of the activity curves, which assesses whether 2 circular distributions (activity periods converted to radians) are statistically different. This test is appropriate for circular data because it accounts for the periodic nature of the data, avoiding biases that linear statistical methods might introduce. This method ensured a valid comparison of the activity distributions between different species and across various time points. The homogeneity of the activity curves was compared using the “watson.wheeler.test” function from the “circilar” package (Agostinelli and Lund 2024). The test enables comparison of homogeneity on 2 or more samples of circular data. If more than 2 samples were compared and the test result was statistically significant (*P* < 0.05), a Watson–Wheeler test was also performed to determine differences between specific pairs. The resulting *P*-value was adjusted using the Bonferroni method.

The influence of the presence of selected variables (binomial distribution) on the occurrence of roe deer activities (binomial distribution) was evaluated using the Wald test, implemented in the function “glm” (family = binomial) in the package “stats” (R Core Team 2022). Generalized linear models (GLMs) are particularly suitable for binary data, as they provide a flexible framework for modeling the relationship between dependent and independent variables without assuming normality. The Wald test provided a rigorous method for hypothesis testing regarding the effects of these variables on roe deer activity.

Akaike information criterion (AIC) was used as a measure of model fit (lower value indicates better model fit). Differences in the duration of specific activities (foraging, vigilance, movement) depending on the selected variables were tested using the Mann–Whitney *U* test in the “stats” package (categorical variable with 2 groups) or, in the case of categorical variables with 3 or more levels, using the Kruskal–Wallis chi square test with Conover post hoc in the “PMCMRplus” package (Pohlert 2022).

## Results

During the 1-year research period, 702 photo records were made in the 3 research areas (Table 2). In Area A, there were 63 human recordings, 136 roe deer recordings, and 4 wolf recordings in the meadow habitat. In the young forest habitat, there were 126 human recordings and 4 wolf recordings; data for roe deer were not recorded. In Area B, the meadow habitat recorded 64 humans and 91 roe deer, with no wolf sightings. In the young forest habitat, there were 32 human recordings and 5 wolf recordings; data for roe deer were not recorded. In Area C (wolf territory), the meadow habitat recorded 21 humans, 86 roe deer, and 4 wolves. In the young forest habitat, there were 1 human recording, 38 roe deer recordings, and 27 wolf recordings. No lynx was recorded on any of the cameras.

**Table 2.** The number of camera trap recordings collected from 11 August 2016, to 11 August 2017, in 3 research areas (A, B, and C) across 2 habitats (M and YF) for 3 species: humans, roe deer, and wolves.

Research area	Habitat	Human	Roe deer	Wolf
A	M	63	136	4
A	YF	126		4
B	M	64	91	
B	YF	32		5
C	M	21	86	4
C	YF	1	38	27

M, meadow; YF, young forest.

**Figure 3:**
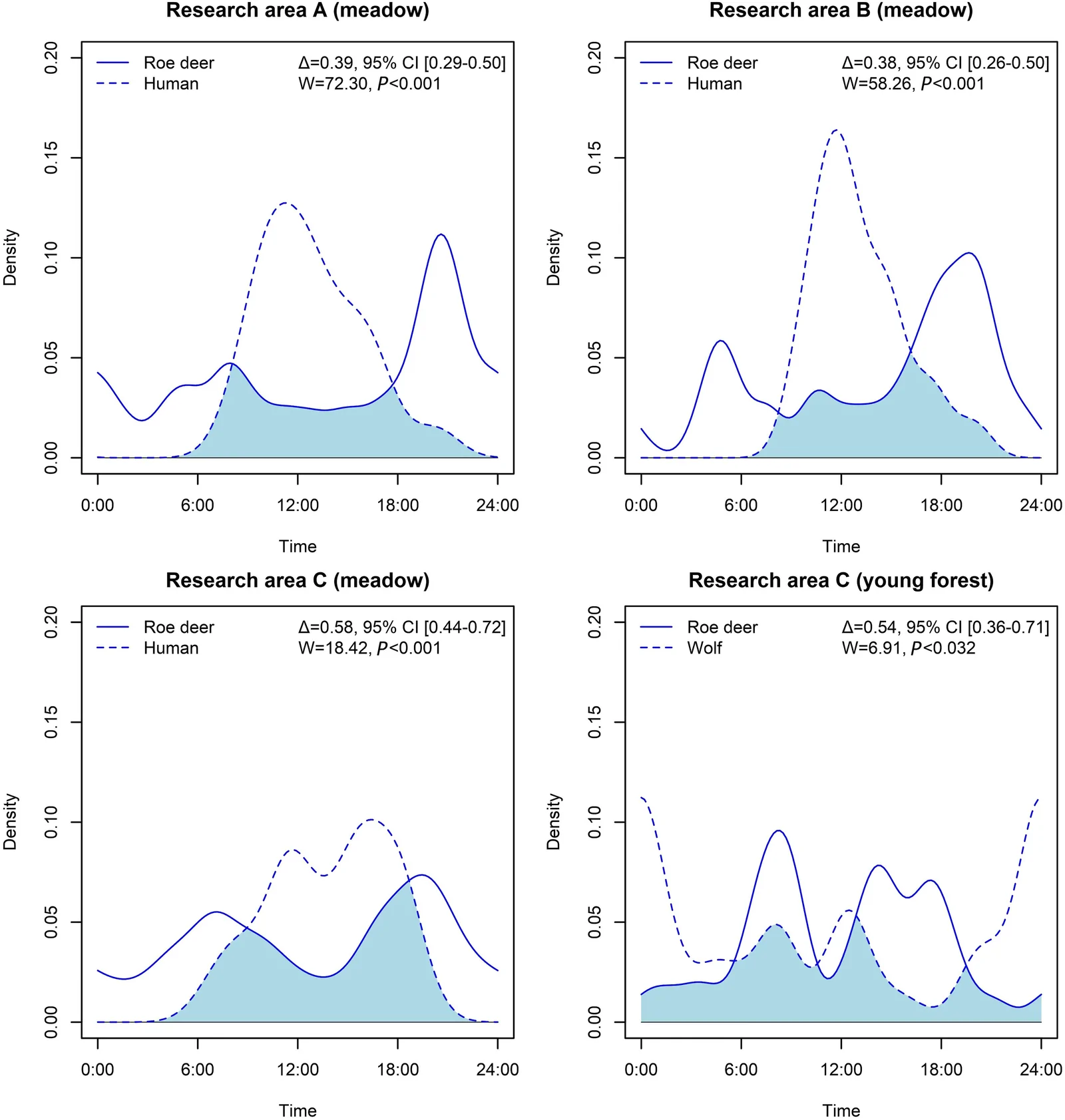
Comparison of roe deer activity with human and wolf activity throughout the day. Graphs show the overlap factor Δ [95% CI] and the result of the Watson–Wheeler test. The figures include all possible comparisons meeting the condition of having more than 5 recordings in each category (research area [i.e., A, B, C] × environment [i.e., meadow {M} or young forest {YF}]).

In meadows, the activity of roe deer was characterized by 2 daily peaks: the first in the morning (lower) and the second in the evening (higher), while peak human activity in the meadow occurred in the afternoon (Fig. 3). The daily activity of roe deer in the meadow differed significantly (*W* = 11.37, *P* = 0.023) but only between Areas A and B, with an overlap coefficient of Δ = 0.77, 95% CI [0.67–0.85]. Human daily activity showed no significant differences in the meadow category between the areas. The daily activity of roe deer in Area C differed significantly between the meadow and the young forest (*W* = 8.50, degrees of freedom [df] = 2, *P*-value = 0.014), with an overlap coefficient of Δ = 0.70, 95% CI [0.56–0.83]. The overlap coefficient of the deer-human activity curve in the meadow ranged from Δ = 0.38, 95% CI [0.26–0.50] (study area B) to Δ = 0.58, 95% CI [0.43–0.73] (Study Area C). There were also 2 peaks of roe deer activity in young forests, with the first (higher) in the morning. The second young forest activity peak of roe deer is more extended in time and starts earlier than in the meadow, after a short but strong afternoon decrease of roe deer activity in the young forest.

Daily wolf activity in the young forest showed 2 peaks, with the first peak at midnight and the second, lower peak at midday. Despite the activity peaks of roe deer and wolf occurring at different times, the overlap coefficient of their activity patterns in the young forest of Study Area C was Δ = 0.54, 95% CI [0.38–0.71]. The overlap coefficient of the activity curve of roe deer in the meadow and young forest was Δ = 0.71, 95% CI [0.58–0.84], with the samples not equally distributed (*W* = 8.42, *P* = 0.015). The overlap coefficient of the activity curves of roe deer in young forest and humans in the meadow was Δ = 0.70, 95% CI [0.53–0.85], with samples equally distributed (*W* = 2.4821, *P* = 0.2891). The potential impact of the overlap between the daily activity patterns of wolves and humans during the day was not compared due to an insufficient sample size.

Three main types of roe deer behavior were distinguished in the collected images: vigilance, foraging, and movement (Table 3). Vigilance was found in 116 recordings (33%), and none of the analyzed variables had a significant effect on the occurrence of this behavior (based on the Wald's test, *P* = 0.05). Foraging was found in 134 recordings (38%). Foraging was significantly influenced by 4 factors: 1) study area (A vs. C [*Z* = 3.728, *P* < 0.001, OR = 2.71] and B vs. C [*Z* = 2.130, *P* = 0.033, OR = 1.88] AIC = 458.19); 2) season (spring vs. winter [*Z* = 2.532, *P* = 0.011, OR = 2.50] AIC = 468.0); 3) time of day (night vs. dusk [*Z* = 2.074, *P* = 0.038, OR = 2.02] AIC = 470.41); and 4) habitat (young forest vs. meadow [*Z* = −2.550, *P* = 0.011, OR = 0.33] AIC = 463.03). Movement, which was detected in 235 records (67%), was significantly affected by: 1) study area (A vs. C [*Z* = −2.769, *P* = 0.006, OR = 0.46] and B vs. C [*Z* = −3.549, *P* < 0.001, OR = 0.34] AIC = 437.07) and 2) habitat (young forest vs. meadow [*Z* = 3.076, *P* = 0.002, OR = 6.59] AIC = 434.4).

**Table 3.** The impact of selected variables on the probability of occurrence of a roe deer behavior by research area (A, B, and C), habitat (M and YF), sex (male and female), time of day (day, night, dawn, and dusk), and season (autumn, summer, spring, and winter), including proportions of behaviors (vigilance, foraging, and movement behaviors).

Variables	Number of videos	Contribution videos with		
		Vigilance	Foraging	Movement
Research area				
A	136	0.32	0.49	0.63
B	91	0.30	0.40	0.56
C	124	0.36	0.26	0.79
Habitat				
M	313	0.30	0.36	0.57
YF	38	0.32	0.18	0.92
Sex				
Female	269	0.33	0.38	0.69
Male	82	0.32	0.40	0.61
Time				
Day	141	0.36	0.38	0.64
Night	68	0.31	0.47	0.63
Dawn	57	0.32	0.39	0.75
Dusk	85	0.31	0.31	0.69
Season				
Autumn	38	0.37	0.37	0.68
Summer	165	0.36	0.39	0.69
Spring	86	0.30	0.47	0.59
Winter	62	0.26	0.26	0.71

M, meadow; YF, young forest.

**Figure 4:**
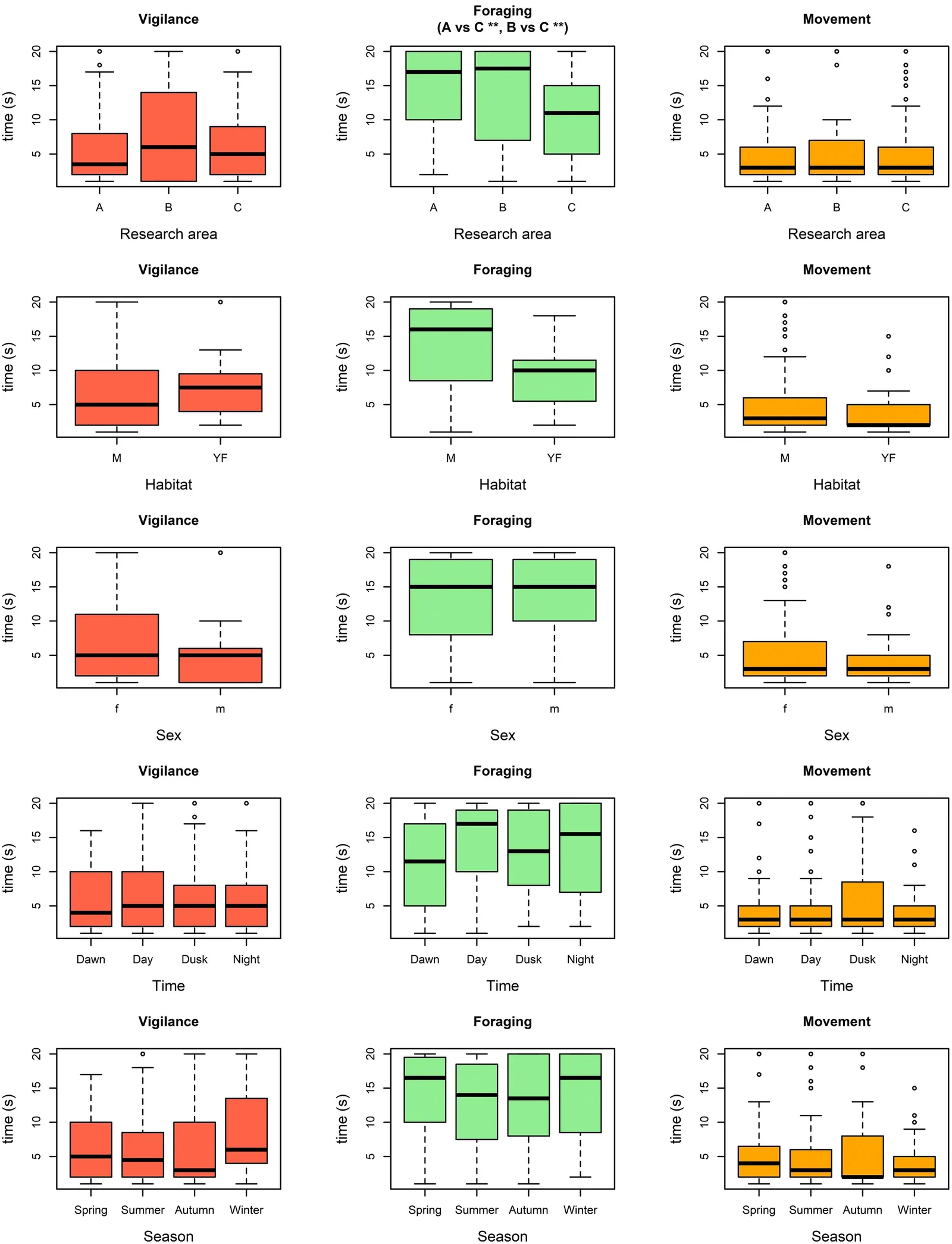
The influence of selected variables such as research area (a, b, and c); habitat (meadow [M] and young forest [YF]); sex: (M and F); and time of day divided into day, night, dawn, and dusk. And season divided into autumn, summer, spring, and winter on the duration of individual deer behaviors such as vigilance, feeding, and movement. Groups that differ statistically at *P* < 0.05 are indicated in parentheses. The significance of differences for 3 or more independent groups was determined using the Kruskal–Wallis chi square test with Conover post hoc (*P* < 0.05), and for 2 groups using the Mann–Whitney *U* test.

The duration of each behavior differed statistically between sites only for foraging (Kruskal–Wallis chi square test = 11.566, *P* = 0.003), with significant differences between areas A vs. C and B vs. C (Fig. 4). The difference in foraging duration between young forest and meadow narrowly failed to be significant (U = 634, *P* = 0.057).

**Figure 5:**
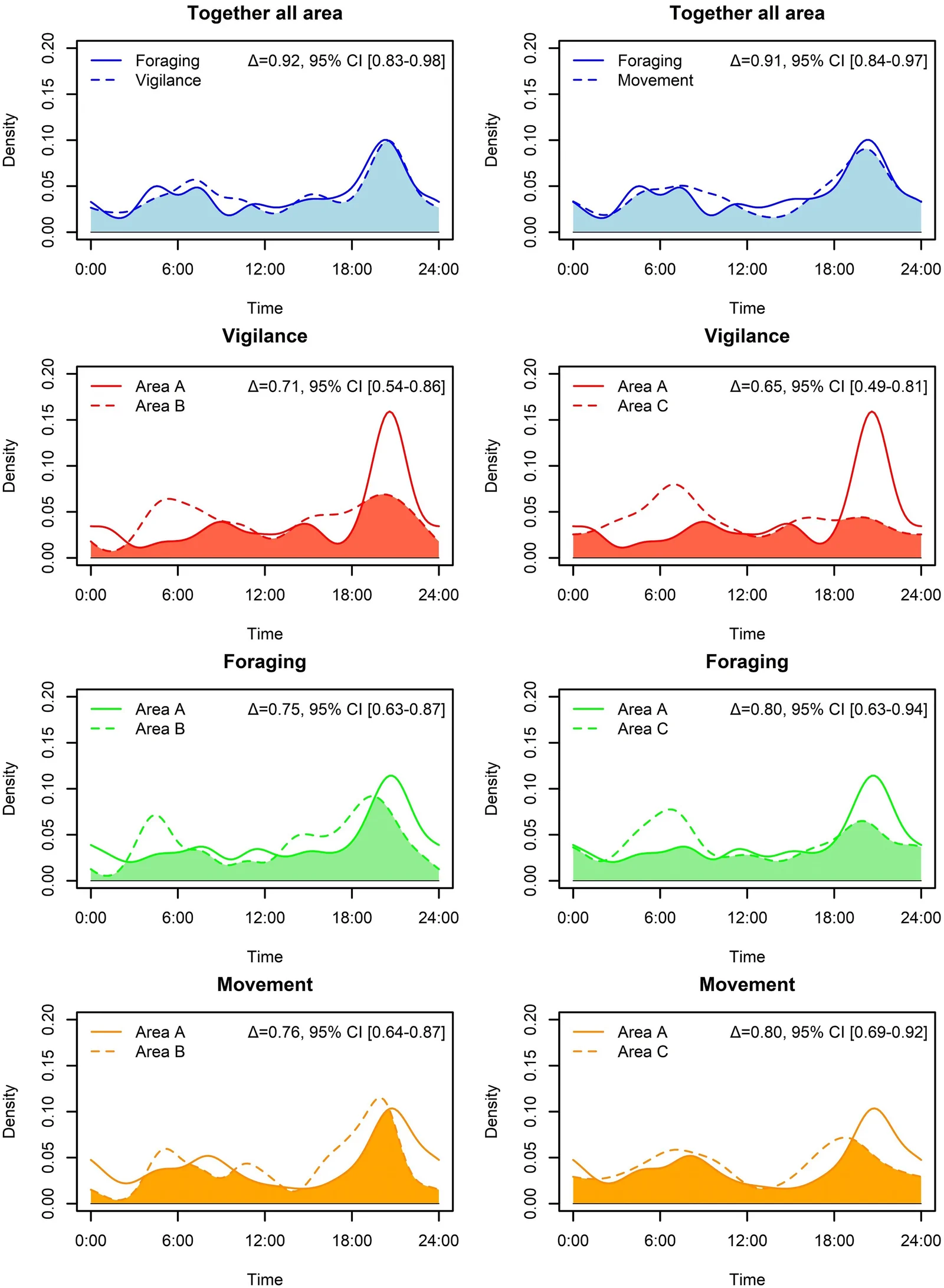
Comparison of various forms of roe deer activity throughout the day in meadows. Graphs show the overlap factor Δ [95% CI].

The coefficient of activity curves for roe deer in meadows (for all research areas combined) show that vigilance and movement have Δ = 0.91 (95% CI [0.84–0.96]). The differences between the 3 types of activity were not significant (*W* = 0.48, *P* = 0.98). Similar results were obtained comparing foraging and vigilance (Δ = 0.92) and foraging and movement (Δ = 0.91) (Fig. 5). The coefficient of activity curves for roe deer vigilance in meadows is highest comparing Areas B and C (Δ = 0.85, 95% CI [0.68–0.97]) but the differences were not statistically significant (*W* = 8.77, *P* = 0.067). For foraging, the coefficient of activity curve for roe deer in meadows ranged from Δ = 0.75 (Areas A and B) to Δ = 0.80 (Areas A and C). Coefficient of activity for research Areas B and C is Δ = 0.77 (95% CI [0.61–0.91]). The differences in this activity were not statistically significant between the areas (*W* = 4.05, *P* = 0.399). For movement, the coefficient of activity curve for roe deer in meadows is highest for Areas B and C (Δ=0.81, 95% CI [0.69–0.92]). The differences in this activity were statistically significant (*W* = 10.06, *P* = 0.039) and occurred between the pair of Areas: A versus B. The proportions of recordings in relation to various factors are presented in the Supplementary Tables S1–S4.

## Discussion

Predation risk by wolves varies spatially and temporally, so deer must use spatial avoidance and movement strategies to reduce encounter rates. Wolves actively hunt and can adapt to the behavior of their prey, so roe deer must respond flexibly, often increasing their movements to avoid detection or pursuit (Petridou et al. 2023). Roe deer have co-evolved with wolves, resulting in finely tuned behavior against predators. In contrast to wolves, human activities usually follow predictable temporal patterns. For example, roe deer can adapt by limiting their activities to low-risk periods (Pagon et al. 2013). Unlike wolves, humans are not direct predators in most cases. However, as some human activities (such as hunting) pose a lethal risk, deer use a general temporal avoidance strategy (Sönnichsen et al. 2013). Human disturbance is often spatially static (e.g., trails) compared with mobile predators such as wolves, so temporal avoidance is the more effective strategy (Bonnot et al. 2013).

Roe deer in area C, characterized by a high risk of wolf presence, exhibited increased movement. However, vigilance was not higher when wolf-risk was greater. The lack of differences in vigilance between areas frequented by humans and wolves leads to some difficulties in determining which species is more stressful to roe deer. One reason no difference between reactions to humans and wolves was detected might be that roe deer react with increased vigilance when coming directly into contact with wolf scent. For example, studies on red deer (*C. elaphus*) demonstrated increased vigilance when exposed to predator feces, including that of wolves (Lung and Childress 2007; Kuijper et al. 2014). Such an experiment was not included in our study. In case of contact with lynx urine, another predator, deer become highly vigilant (Eccard et al. 2017). Permanent vigilance by roe deer is an unprofitable behavior. Increased movement, often a stress-induced response, is one of the behaviors ungulates like roe deer use to avoid predation, which in turn affects lower elements of the trophic cascade (Frair et al. 2005). Vigilance typically involves stationary scanning of the environment for threats, which comes at the cost of reduced mobility and foraging time (Lung and Childress 2007). In high-predation environments, roe deer might prioritize movement over vigilance as a survival strategy. By moving more frequently, they reduce their time spent in any one location, which could make it harder for wolves to ambush them (Benhaiem et al. 2008). Roe deer in areas with high wolf pressure may become habituated to the presence of wolves and adjust their behavior accordingly. Instead of constant heightened vigilance, which can be energetically costly, they may adopt movement as a more sustainable antipredator strategy (Kuijper et al. 2014). Over time, these animals may have learned that staying on the move is a more effective way to avoid predation than standing still and scanning for predators. Roe deer might rely on habitat features (e.g., dense vegetation) that reduce the need for visual vigilance (Lone et al. 2015). In such environments, movement may serve to position them in safer areas where the risk of wolf encounters is lower. It is worth said here that roe deer were recorded in the young forest only and exclusively in an area with a high risk of wolf predation. For instance, they might move into denser forest areas after detecting predator signs, relying more on concealment to avoid detection rather than visual scanning. In the meadows of Area C with a high-predation risk, roe deer may have had to spend more time searching for higher-value food, which may have increased the intensity of movement. However, roe deer preference for types of habitat is most influenced by forest structure, that is, canopy openness and tree species richness, rather than food abundance (Moser et al. 2006). In BTNP, Scots pine is the dominant overstory species, and the age structure of stands is not highly diversified. As shown by Churski et al. (2021), predation risk may affect the species composition of ungulate diets. The increased movement of deer in predator-rich locations may also be caused by the search for better quality/higher calorific value food to compensate for energy loss from movement. Humans may cause more stress in areas of high human pressure than does the presence of wolves where they occur (Zbyryt et al. 2018). High wolf pressure combined with individual hunting can significantly reduce the feeding time of roe deer due to the increased perceived predation risk. In areas with frequent predator presence, roe deer must allocate more time to antipredator behaviors, such as movement and habitat selection, reducing the time available for foraging (Benhaiem et al. 2008). Additionally, roe deer may be forced to feed in suboptimal, more concealed habitats to reduce predation risk, which can limit access to high-quality forage, further compounding the reduction in feeding time (Lone et al. 2015). Thus, increased time spent searching for food is due to the smaller area deer confine themselves to in the presence of predators, resulting in consumption of the “closest” food and precluding the possibility of searching a larger area for better quality food.

In meadows in Areas A and B, roe deer modified daily activities to temporarily avoid humans. More specifically, roe deer avoid humans in meadows despite this environment's attractiveness for foraging, regardless of the form of human disturbance they are dealing with (i.e., for both recreation without hunting in Area A and for heavy hunting pressure in Area B). This is surprising, as hunting would be expected to have a stronger influence on roe deer behavior than recreation. But hunting tends to be concentrated over shorter, defined periods, leading to intense but temporary stress on deer (Viela et al. 2020). Thus, highly intense recreation in BTNP could have an effect similar to hunting on roe deer activity. Recreation has a scaring effect on ungulate behavior that reduces browsing (van Beeck Calkoen et al. 2022). This is especially true in BTNP, where, unlike in other national parks in Poland, people are permitted to move off of tourist trails. Avoidance of humans may also affect the duration of foraging, as mentioned above. Deer will consume the nearest browse without searching for browse of better quality. This is because the time deer spend foraging is shorter than in places with lower levels of human disturbance (Bonnot et al. 2020).

In the area with high-predation risk by wolves and, at the same time, low pressure by humans (Area C), roe deer were not observed avoiding humans in meadows. This may be due to several factors. It is perhaps possible that limited human disturbance is less important when roe deer are strongly at risk of predation by wolves. However, the small number of individuals in this study made detection of statistical significance difficult. Finally, just as deer do not avoid wolves in areas with good cover, such locations are perceived as shelter rather than risk, even in the presence of predators. Young forests serve as resting places for wolves during the day (Bojarska et al. 2021), the peak of activity in these places was at noon and second in the morning. The peak of hunting activity occurs at dawn and dusk (Tharenkauf et al. 2003). This may also be the reason for the lack of statistical significance. The low number of wolf–human records during the day suggests that such interactions are not frequent enough to have introduced potential bias into the present results.

Roe deer and wolves both use cover to hide from humans. If they hide from humans and rest at the same time, then wolves are clearly not a threat to the deer because deer are busy avoiding their common enemy, that is, humans (Bojarska et al. 2021). Moreover, ungulates such as roe deer are more stressed by the presence of humans than by predators because they are evolutionarily adapted to avoid wolves (Zbyryt et al. 2018). Our results may confirm to some extent that roe deer, due to high risk of wolf predation, may choose covers more often as a place of safety, not only females during the rearing period (Bongi et al. 2008). On the other hand, we were unable to confirm in any way the temporal avoidance of wolves by deer or temporal synchronization as proven in other studies (Rossa et al. 2021; Petridou et al. 2023).

A shortcoming of this study was the use of camera traps. However, we believe that camera observation of the effects of factors such as various types of anthropogenic pressure and wolf predation on roe deer behavior is valuable. In particular, this study was conducted at a time when large forest complexes in Poland had only partially been recolonized by wolves. This provided the rare opportunity to analyze deer behavior under varying levels of predation risk and human pressure. Another valuable feature of this study was the ability to evaluate the responses of roe deer to humans and wolves in the absence of lynx, which is elsewhere a major predator of roe deer. The difference in roe deer responses to wolves and humans stems from the distinct nature of these threats. Wolf predation is a challenge that requires increased movement to minimize risk, while human disturbances are a pressure mitigated through temporal avoidance. These strategies reflect the roe deer's behavioral plasticity but also underscore the costs of adapting to multiple stressors. The findings highlight how predators and human activities differently shape roe deer behavior, offering valuable insights for conservation. Altered movement patterns in response to wolves suggest potential physiological consequences and increased energetic costs in roe deer, while the temporal avoidance of human activity emphasizes the need to minimize disturbances. By considering these behavioral responses, conservation strategies can better support the coexistence of wildlife and human activities.

## Supplementary Material

zoaf059_Supplementary_Data

## Data Availability

The data are available upon request.

## References

[zoaf059-B1] Agostinelli C, Lund U. 2024. R package ‘circular’: circular statistics (version 0.5-1). [Accessed 2024 May 20]. Available from: https://CRAN.R-project.org/package=circular

[zoaf059-B2] Ausband DE , 2021. Wolf use of humanmade objects during pup-rearing. Anim Behav Cogn 8(3):405–414.

[zoaf059-B3] Ausilio G, Sand H, Månsson J, Mathisen KM, Wikenros C, 2021. Ecological effects of wolves in anthropogenic landscapes: the potential for trophic cascades is context-dependent. Front Ecol Evol 8:1–12.

[zoaf059-B4] Benhaiem S, Delon M, Lourtet B, Cargnelutti B, Aulagnier S et al, 2008. Hunting increases vigilance levels in roe deer and modifies feeding site selection. Anim Behav 76(3):611–618.

[zoaf059-B5] Bojarska K, Maugeri L, Kuehn R, Król W, Theuerkauf J et al, 2021. Wolves under cover: the importance of human-related factors in resting site selection in a commercial forest. For Ecol Manag 497:119511.

[zoaf059-B6] Bongi P, Ciuti S, Grignolio S, Del Frate M, Simi S et al, 2008. Anti-predator behaviour, space use and habitat selection in female roe deer during the fawning season in a wolf area. J Zool 276(3):242–251.

[zoaf059-B7] Bonnot NC, Couriot O, Berger A, Cagnacci F, Ciuti S et al, 2020. Fear of the dark? Contrasting impacts of humans versus lynx on diel activity of roe deer across Europe. J Anim Ecol 89(1):132–145. 31799691 10.1111/1365-2656.13161

[zoaf059-B8] Bonnot NC, Morellet N, Verheyden H, Cargnelutti B, Lourtet B et al, 2013. Habitat use under predation risk: hunting, roads and human dwellings influence the spatial behaviour of roe deer. Eur J Wildl Res 59(2):185–193.

[zoaf059-B9] Burbaite L, Csányi S, 2009. Roe deer population and harvest changes in Europe. Estonian J Ecol 58(3):169–180.

[zoaf059-B10] Carbillet J, Rey B, Palme R, Morellet N, Bonnot N et al, 2020. Under cover of the night: context-dependency of anthropogenic disturbance on stress levels of wild roe deer *Capreolus capreolus*. Conserv Physiol 8(1):1–11. 10.1093/conphys/coaa086 PMC7507870 32995004

[zoaf059-B11] Chapron G, Kaczensky P, Linnell JDC, Von Arx M, Huber D et al, 2014. Recovery of large carnivores in Europe's modern human-dominated landscapes. Science 346(6216):1517–1519. 25525247 10.1126/science.1257553

[zoaf059-B12] Churski M, Spitzer R, Coissac E, Taberlet P, Lescinskaite J et al, 2021. How do forest management and wolf space-use affect diet composition of the wolf's main prey, the red deer versus a non-prey species, the European bison? For Ecol Manag 479:118620.

[zoaf059-B13] Creel S, Christianson D, 2009. Wolf presence and increased willow consumption by Yellowstone elk: implications for trophic cascades. Ecology 90(9):2454–2466. 19769124 10.1890/08-2017.1

[zoaf059-B14] Creel S, Winnie JA, 2005. Responses of elk herd size to fine-scale spatial and temporal variation in the risk of predation by wolves. Anim Behav 69(5):1181–1189.

[zoaf059-B15] Eccard JA, Meißner JK, Heurich M, 2017. European roe deer increase vigilance when faced with immediate predation risk by Eurasian lynx. Ethology 123(1):30–40.

[zoaf059-B16] Frair JL, Merrill EH, Visscher DR, Fortin D, Beyer HL et al, 2005. Scales of movement by elk (*Cervus elaphus*) in response to heterogeneity in forage resources and predation risk. Landsc Ecol 20(3):273–287.

[zoaf059-B17] Gerber N, Riesch F, Bojarska K, Zetsche M, Rohwer NK et al, 2024. Do recolonising wolves trigger non-consumptive effects in European ecosystems? A review of evidence. Wildl Biol 2024(6):e01229.

[zoaf059-B18] Halofsky JS, Ripple WJ, 2008. Fine-scale predation risk on elk after wolf reintroduction in Yellowstone National Park, USA. Oecologia 155(4):869–877. 18224339 10.1007/s00442-007-0956-z

[zoaf059-B19] Hebblewhite M, Merrill EH, 2007. Multiscale wolf predation risk for elk: does migration reduce risk? Oecologia 152(2):377–387. 17287955 10.1007/s00442-007-0661-y

[zoaf059-B20] Hušek J, Boudreau MR, Panek M, 2021. Hunter estimates of game density as a simple and efficient source of information for population monitoring: a comparison to targeted survey methods. PLoS One 16(8):e0256580. 34424923 10.1371/journal.pone.0256580 PMC8382201

[zoaf059-B21] Jasińska KD, Jackowiak M, Gryz J, Bijak S, Szyc K et al, 2021. Occurrence and activity of roe deer in urban forests of Warsaw. Diversity (Basel) 3(1):7913.

[zoaf059-B22] Jones O, Wang J, 2010. COLONY a program for parentage and sibship inference from multilocus genotype data. Mol Ecol Resour 10(3):551–555. 21565056 10.1111/j.1755-0998.2009.02787.x

[zoaf059-B23] Kalinowski ST, Taper ML, Marshall TC, 2007. Revising how the computer program CERVUS accommodates genotyping error increases success in paternity assignment. Mol Ecol 16(5):1099–1106. 17305863 10.1111/j.1365-294X.2007.03089.x

[zoaf059-B24] Kondracki J , 2002. Regional Geography of Poland. Warsaw: PWN Scientific Publishers, 79–86.

[zoaf059-B25] Kuijper DPJ, Kleine CD, Churski M, Hooft PV, Bubnicki J, 2013. Landscape of fear in Europe : wolves affect spatial patterns of ungulate browsing in Białowiez a Primeval Forest, Poland. Ecography 36(12):1263–1275.

[zoaf059-B26] Kuijper DPJ, Verwijmeren M, Churski M, Zbyryt A, Schmidt K et al, 2014. What cues do ungulates use to assess predation risk in dense temperate forests? PLoS One 9(1):e84607. 24404177 10.1371/journal.pone.0084607 PMC3880296

[zoaf059-B27] Laundre JW, Hernandez L, Ripple WJ, 2010. The landscape of fear: ecological implications of being afraid. Open Ecol J 3(3):1–7.

[zoaf059-B28] Linnell JDC, Cretois B, Nilsen EB, Rolandsen CM, Solberg EJ et al, 2020. The challenges and opportunities of coexisting with wild ungulates in the human-dominated landscapes of Europe's Anthropocene. Biol Conserv 244:108500.

[zoaf059-B29] Lone K, Loe LE, Meisingset EL, Stamnes I, Mysterud A, 2015. An adaptive behavioural response to hunting: surviving male red deer shift habitat at the onset of the hunting season. Anim Behav 102:127–138.

[zoaf059-B30] Loosen AE, Devineau O, Zimmermann B, Cromsigt JPGM, Pfeffer SE et al, 2021. Roads, forestry, and wolves interact to drive moose browsing behavior in Scandinavia. Ecosphere 12(1):e03358.

[zoaf059-B31] Lovari S, Herrero J, Masseti M, Ambarli H, Lorenzini R et al, 2016. Capreolus capreolus. *The IUCN Red List of Threatened Species* **2016**: e.T42395A22161386.

[zoaf059-B32] Lung MA, Childress MJ, 2007. The influence of conspecifics and predation risk on the vigilance of elk (*Cervus elaphus*) in Yellowstone National Park. Behav Ecol 18(1):12–20.

[zoaf059-B33] Lynch M, Ritland K, 1999. Estimation of pairwise relatedness with molecular markers. Genetics 152(4):1753–1766. 10430599 10.1093/genetics/152.4.1753 PMC1460714

[zoaf059-B34] Mols B, Lambers E, Cromsigt JPGM, Kuijper DPJ, Smit C, 2022. Recreation and hunting differentially affect deer behaviour and sapling performance. Oikos 2022(1):1–13.

[zoaf059-B35] Moser B, Schütz M, Hindenlang KE, 2006. Importance of alternative food resources for browsing by roe deer on deciduous trees: the role of food availability and species quality. For Ecol Manag 226(1–3):248–255.

[zoaf059-B36] Mpemba H, Fan Y, Macleod KJ, Wen D, Jiang G, 2019. The effect of novel and familiar predator cues on prey vigilance and foraging behaviors in the greater Khingan mountains, Inner Mongolia, China. Appl Ecol Environ Res 17(4):8219–8234.

[zoaf059-B37] Mysłajek R, Kwiatkowska I, Diserens TA, Haidt A, Nowak S, 2019. Occurrence of the Eurasian lynx in western Poland after two decades of strict protection. Cat News 69:12–14.

[zoaf059-B38] Mysłajek RW, Tracz M, Tracz M, Tomczak P, Szewczyk M et al, 2018. Spatial organization in wolves Canis lupus recolonizing north-west Poland: Large territories at low population density. Behaviour 92:37–44. 10.1016/j.mambio.2018.01.006.

[zoaf059-B39] Nowak S, Mysłajek RW, 2016. Wolf recovery and population dynamics in Western Poland, 2001–2012. Mammal Res 61(2):83–98.

[zoaf059-B40] Nowak S, Mysłajek RW, Kłosińska A, Gabryś G, 2011. Diet and prey selection of wolves (*Canis lupus*) recolonising Western and Central Poland. Mamm Biol 76(6):709–715.

[zoaf059-B41] Nowak S, Mysłajek RW, Szewczyk M, Tomczak P, Borowik T et al, 2017. Sedentary but not dispersing wolves *Canis lupus* recolonizing western Poland (2001–2016) conform to the predictions of a habitat suitability model. Divers Distrib 23(11):1353–1364.

[zoaf059-B42] Pagon N, Grignolio S, Pipia A, Bongi P, Bertolucci C et al, 2013. Seasonal variation of activity patterns in roe deer in a temperate forested area. Chronobiol Int 30(6):772–785. 23738905 10.3109/07420528.2013.765887

[zoaf059-B43] Peakall R, Smouse PE, 2012. Genalex 6.5: genetic analysis in Excel. Population genetic software for teaching and research—an update. Bioinformatics 28(19):2537–2539. 22820204 10.1093/bioinformatics/bts460 PMC3463245

[zoaf059-B44] Petridou M, Benson JF, Gimenez O, Kati V, 2023. Spatiotemporal patterns of wolves, and sympatric predators and prey relative to human disturbance in Northwestern Greece. Diversity (Basel) 15(2):184.

[zoaf059-B45] Picardi S, Basille M, Peters W, Ponciano JM, Boitani L et al, 2019. Movement responses of roe deer to hunting risk. J Wildl Manag 83(1):43–51.

[zoaf059-B46] Pohlert T . 2022. PMCMRplus: calculate pairwise multiple comparisons of mean rank sums extended_. R package version 1.9.6. [Accessed 2024 May 20]. Available from: https://CRAN.R-project.org/package=PMCMRplus

[zoaf059-B47] Proudman NJ, Churski M, Bubnicki JW, Nilsson JÅ, Kuijper DPJ, 2021. Red deer allocate vigilance differently in response to spatio-temporal patterns of risk from human hunters and wolves. Wildl Res 48(2):163–174.

[zoaf059-B48] Raynor JL, Grainger CA, Parker DP, 2021. Wolves make roadways safer, generating large economic returns to predator conservation. Proc Natl Acad Sci U S A 118(22):e2023251118. 34031245 10.1073/pnas.2023251118 PMC8179214

[zoaf059-B49] R Core Team , 2022. R: A Language and Environment for Statistical Computing Vienna, Austria: R Foundation for Statistical Computing.

[zoaf059-B50] Ridout MS, Linkie M, 2009. Estimating overlap of daily activity patterns from camera trap data. J Agric Biol Environ Stat 14(3):322–337.

[zoaf059-B51] Rodríguez-Recio M, Wikenros C, Zimmermann B, Sand H, 2022. Rewilding by wolf recolonisation, consequences for ungulate populations and game hunting. Biology (Basel) 11(2):317. 35205183 10.3390/biology11020317 PMC8869524

[zoaf059-B52] Rossa M, Lovari S, Ferretti F, 2021. Spatiotemporal patterns of wolf, mesocarnivores and prey in a Mediterranean area. Behav Ecol Sociobiol 75(2):32.

[zoaf059-B53] Sönnichsen L, Bokje M, Marchal J, Hofer H, Jędrzejewska B et al, 2013. Behavioural responses of European roe deer to temporal variation in predation risk. Ethology 119(3):233–243.

[zoaf059-B54] Stępniak KM, Niedźwiecka N, Szewczyk M, Mysłajek RW, 2020. Scent marking in wolves *Canis lupus* inhabiting managed lowland forests in Poland. Mammal Res 65(4):629–638.

[zoaf059-B55] Szewczyk M, Nowak C, Hulva P, Mergeay J, Stronen AV et al, 2021. Genetic support for the current discrete conservation unit of the Central European wolf population. Wildl Biol 2021(2):1–7.

[zoaf059-B56] Szewczyk M, Nowak S, Niedźwiecka N, Hulva P, Špinkytė-Bačkaitienė R et al, 2019. Dynamic range expansion leads to establishment of a new, genetically distinct wolf population in Central Europe. Sci Rep 9(1):19003. 31831858 10.1038/s41598-019-55273-w PMC6908625

[zoaf059-B57] Theuerkauf J, Jȩdrzejewski W, Schmidt K, Okarma H, Ruczyński I et al, 2003. Daily patterns and duration of wolf activity in the Białowieza Forest, Poland. J Mammal 84(1):243–253.

[zoaf059-B58] Thomas K , 2011. A history of camera trapping. In: Kucera TE, Barrett RH, editors. Camera Traps in Animal Ecology Tokyo: Springer, 9–26.

[zoaf059-B59] Tobler MW, Carrillo-Percastegui SE, Leite Pitman R, Mares R, Powell G, 2008. An evaluation of camera traps for inventorying large- and medium-sized terrestrial rainforest mammals. Anim Conserv 11(3):169–178.

[zoaf059-B60] Torretta E, Caviglia L, Serafini M, Meriggi A, 2018. Wolf predation on wild ungulates: how slope and habitat cover influence the localization of kill sites. Curr Zool 64(3):271–275. 30403201 10.1093/cz/zox031 PMC6007434

[zoaf059-B61] Torretta E., Mosini A., Piana M., Tirozzi P., Serafini M., Puopolo F., et al Time partitioning in mesocarnivore communities from different habitats of NW Italy: insights into martens’ competitive abilities. Behaviour 2017;154(2):241–266. 10.1163/1568539X-00003420

[zoaf059-B62] van Beeck Calkoen STS, Deis MH, Oeser J, Kuijper DPJ, Heurich M, 2022. Humans rather than Eurasian lynx (Lynx lynx) shape ungulate browsing patterns in a temperate forest. Ecosphere 13(2):1–19.38988721

[zoaf059-B63] van Beeck Calkoen STS, Kuijper DPJ, Sand H, Singh NJ, van Wieren SE et al, 2018. Does wolf presence reduce moose browsing intensity in young forest plantations? Ecography 41(11):1776–1787.

[zoaf059-B64] Vilela S, Alves da Silva A, Palme R, Ruckstuhl KE, Sousa JP et al, 2020. Physiological stress reactions in red deer induced by hunting activities. Animals (Basel) 10(6):1003. 32521768 10.3390/ani10061003 PMC7341308

[zoaf059-B65] Winnie J, Creel S, 2007. Sex-specific behavioural responses of elk to spatial and temporal variation in the threat of wolf predation. Anim Behav 73(1):215–225.

[zoaf059-B66] Zbyryt A, Bubnicki JW, Kuijper DPJ, Dehnhard M, Churski M et al, 2018. Do wild ungulates experience higher stress with humans than with large carnivores? Behav Ecol 29(1):19–30.

