# Supplementary material for: Reactions of roe deer to human disturbances and wolf predation in a Polish forest
Source: Curr Zool. 2025 Aug 28;72(4):486–99. doi: 10.1093/cz/zoaf059 (PMC13500159; doi:10.1093/cz/zoaf059)
Supplement: Supplementary file 1 [file zoaf059_supplementary_data.zip › Supplementary table 1.docx]

Supplementary Table 1. Comparison of the types of roe deer activity in the meadow between Research Areas (A, B, C) in different seasons (Spring, Summer, Autumn, Winter). N mean number of recordings (activities compared successively), Δ - overlap coefficient and its confidence interval [95% CI], W – Watson- Wheeler test result, statistically significant differences (*P*<0.05) are marked with an asterisk (*).

| Activity | Research Area | Test | Spring | Summer | Autumn | Winter |
| --- | --- | --- | --- | --- | --- | --- |
|  | All | W | 5.11 | 6.70 | 6.17 | 4.95 |
| Vigilance | A and B | n | 9; 7 | 30; 12 | 3; 2 | 2; 6 |
|  |  | Δ | 0.56 | 0.56 | 0.60 | 0.18 |
|  |  | [95% CI] | 0.27-0.80 | 0.32-0.76 | 0.27-1.07 | 0.03-0.53 |
|  |  |  |  |  |  |  |
|  | A and C | n | 9; 8 | 30; 16 | 3; 6 | 2; 3 |
|  |  | Δ | 0.46 | 0.55 | 0.37 | 0.51 |
|  |  | [95% CI] | 0.18-0.73 | 0.31-0.76 | 0.11-0.70 | 0.14-0.95 |
|  |  |  |  |  |  |  |
|  | B and C | n | 7; 8 | 12; 16 | 2; 6 | 6; 3 |
|  |  | Δ | 0.59 | 0.67 | 0.36 | 0.16 |
|  |  | [95% CI] | 0.28-0.88 | 0.44-0.86 | 0.14-0.70 | 0.04-0.44 |
|  |  |  |  |  |  |  |
|  | All | W | 5.39 | 7.35 | 4.19 | 7.66 |
| Foraging | A and B | n | 20; 12 | 37; 12 | 6; 3 | 3; 9 |
|  |  | Δ | 0.73 | 0.53 | 0.53 | 0.43 |
|  |  | [95% CI] | 0.53-0.92 | 0.33-0.72 | 0.16-0.93 | 0.14-0.77 |
|  |  |  |  |  |  |  |
|  | A and C | n | 20; 7 | 37; 14 | 6; 3 | 3; 1 |
|  |  | Δ | 0.60 | 0.62 | 0.31 | NA |
|  |  | [95% CI] | 0.33-0.85 | 0.40-0.81 | 0.06-0.65 | NA |
|  |  |  |  |  |  |  |
|  | B and C | n | 12; 7 | 12; 14 | 3; 3 | 9; 1 |
|  |  | Δ | 0.45 | 0.57 | 0.40 | NA |
|  |  | [95% CI] | 0.20-0.74 | 0.34-0.78 | 0.10-0.82 | NA |
|  |  |  |  |  |  |  |
|  | All | W | 2.21 | 13.03* | 5.81 | 1.96 |
| Movement | A and B | n | 17; 11 | 53; 29 | 7; 4 | 9; 7 |
|  |  | Δ | 0.67 | 0.67 | 0.22 | 0.83 |
|  |  | [95% CI] | 0.46-0.86 | 0.52-0.81 | 0.01-0.52 | 0.53-1.06 |
|  |  |  |  |  |  |  |
|  | A and C | n | 17; 19 | 53; 23 | 7; 7 | 9; 14 |
|  |  | Δ | 0.74 | 0.68 | 0.62 | 0.58 |
|  |  | [95% CI] | 0.52-0.92 | 0.52-0.83 | 0.32-0.87 | 0.31-0.81 |
|  |  |  |  |  |  |  |
|  | B and C | n | 11; 19 | 29; 23 | 4; 7 | 7; 14 |
|  |  | Δ | 0.53 | 0.73 | 0.13 | 0.63 |
|  |  | [95% CI] | 0.29-0.75 | 0.54-0.88 | 0.01-0.43 | 0.36-0.85 |
|  |  |  |  |  |  |  |
