# Supplementary material for: Reactions of roe deer to human disturbances and wolf predation in a Polish forest
Source: Curr Zool. 2025 Aug 28;72(4):486–99. doi: 10.1093/cz/zoaf059 (PMC13500159; doi:10.1093/cz/zoaf059)
Supplement: Supplementary file 1 [file zoaf059_supplementary_data.zip › Supplementary table 2.docx]

Supplementary Table 2. The total number of recordings in which specific behaviors appeared: Vigilance, Foraging, Movement, depending on the Research Area number.

| Research Area | Vigilance | Foraging | Movement |
| --- | --- | --- | --- |
| A | 44 | 66 | 86 |
| B | 27 | 36 | 51 |
| C | 45 | 32 | 98 |
| Sum | 116 | 134 | 235 |
