# Supplementary material for: Reactions of roe deer to human disturbances and wolf predation in a Polish forest
Source: Curr Zool. 2025 Aug 28;72(4):486–99. doi: 10.1093/cz/zoaf059 (PMC13500159; doi:10.1093/cz/zoaf059)
Supplement: Supplementary file 1 [file zoaf059_supplementary_data.zip › Supplementary table 3.docx]

Supplementary Table 3. The proportion of recordings in which behaviors appeared: Vigilance, Foraging, Movement, depending on the Research Area number (A, B, C), Season (Spring, Summer, Autumn, Winter), Time of Day (Day, Night, Dawn, Dusk), Sex (m, f), and Habitat (YF - Young Forest, M - Meadow).

| Variable | | N videos | Recordings share | | |
| --- | --- | --- | --- | --- | --- |
|  |  |  | Vigilance | Foraging | Movement |
| Research Area | A | 136 | 0,32 | 0,49 | 0,63 |
|  | B | 91 | 0,30 | 0,40 | 0,56 |
|  | C | 124 | 0,36 | 0,26 | 0,79 |
| Habitat | M | 313 | 0,30 | 0,00 | 0,57 |
|  | YF | 38 | 0,32 | 0,18 | 0,92 |
| Sex | f | 269 | 0,33 | 0,38 | 0,69 |
|  | m | 82 | 0,32 | 0,40 | 0,61 |
| Time of Day | Day | 141 | 0,36 | 0,38 | 0,64 |
|  | Night | 68 | 0,31 | 0,47 | 0,63 |
|  | Dawn | 57 | 0,32 | 0,39 | 0,75 |
|  | Dusk | 85 | 0,31 | 0,31 | 0,69 |
| Season | Autumn | 38 | 0,37 | 0,37 | 0,68 |
|  | Summer | 165 | 0,36 | 0,39 | 0,69 |
|  | Spring | 86 | 0,30 | 0,47 | 0,59 |
|  | Winter | 62 | 0,26 | 0,26 | 0,71 |
