# Supplementary material for: Reactions of roe deer to human disturbances and wolf predation in a Polish forest
Source: Curr Zool. 2025 Aug 28;72(4):486–99. doi: 10.1093/cz/zoaf059 (PMC13500159; doi:10.1093/cz/zoaf059)
Supplement: Supplementary file 1 [file zoaf059_supplementary_data.zip › Supplementary table 4.docx]

Supplementary Table 4. The proportion of recordings in which behaviors appeared: Vigilance, Foraging, Movement, depending on the Research Area number (A, B, C) and Habitat (YF - Young Forest, M - Meadow).

| Research Area | Habitat | N Videos | Recordings share | | |
| --- | --- | --- | --- | --- | --- |
|  |  |  | Vigilance | Foraging | Movement |
| A | M | 136 | 0,32 | 0,49 | 0,63 |
| A | YF | 0 | . | . | . |
| B | M | 91 | 0,30 | 0,40 | 0,56 |
| B | YF | 0 | . | . | . |
| C | M | 86 | 0,34 | 0,26 | 0,66 |
| C | YF | 38 | 0,32 | 0,18 | 0,92 |
| C | Sum | 124 | 0,36 | 0,26 | 0,79 |
| Sum |  | 351 | 0,33 | 0,38 | 0,67 |
